# Complement in cancer-associated fibroblasts: sources, regulation and context dependence

**DOI:** 10.3389/fimmu.2026.1996730

**Published:** 2026-10-30

**Authors:** Kaiqi Lei

**Affiliations:** School of Biosciences, University of Sheffield, Sheffield, United Kingdom

**Keywords:** cancer-associated fibroblasts, complement, complement source attribution, fibroblast biology, inflammatory CAF (iCAF), tumour microenvironment

## Abstract

Complement genes recur in the transcriptomes of cancer-associated fibroblasts (CAFs) and have been independently reproduced across pancreatic, breast, oesophageal, head-and-neck and uveal melanoma tumours. This review argues that complement is a real, perturbable functional dimension of fibroblasts, but that four questions central to interpreting it in cancer remain unresolved: which cell contributes how much complement protein in intact tissue; whether production is coupled to functional self-protection; whether the proximal and terminal ends of the cascade are differentially regulated; and whether the malignant secretome, rather than generic stress, is what induces the phenotype. Across studies the same recurrent signature is assigned to at least seven different CAF state labels, so equating complement-positive fibroblasts with inflammatory CAFs (iCAFs) is a naming convention rather than a validated attribution. Trajectory and atlas evidence suggest complement may be a feature already present in normal and early fibroblasts that declines with progression; direct induction and perturbation evidence comes mainly from therapeutic injury, chronic inflammation or already-established CAFs; and no study yet pairs a malignant with a matched non-malignant secretome under a multi-component complement readout. The central message is that the next step is not another transcriptomic atlas but one defined experiment paired malignant and non-malignant inputs on the same fibroblasts, complement quantified at the protein and secretion level, and fibroblast-specific perturbation with a tissue complement readout, that would address all four questions at once.

## 1 Introduction

A recurrent transcriptional signature defines a stable co-expression phenomenon whose underlying mechanism remains to be clarified. Although many studies report enrichment of complement genes in cancer-associated fibroblasts (CAFs), the supporting evidence comes mainly from single-cell and spatial transcriptomics. These studies can identify cells carrying complement transcripts, but they struggle to answer which signals drive complement expression, whether the transcripts are translated into secreted protein, and what fraction of the complement output in multi-cellular tumour tissue CAFs actually contribute (1–3). Integrating the scattered evidence, four distinct questions can be distilled, around which this review is organised (**Figure 1**).

**Figure 1:**
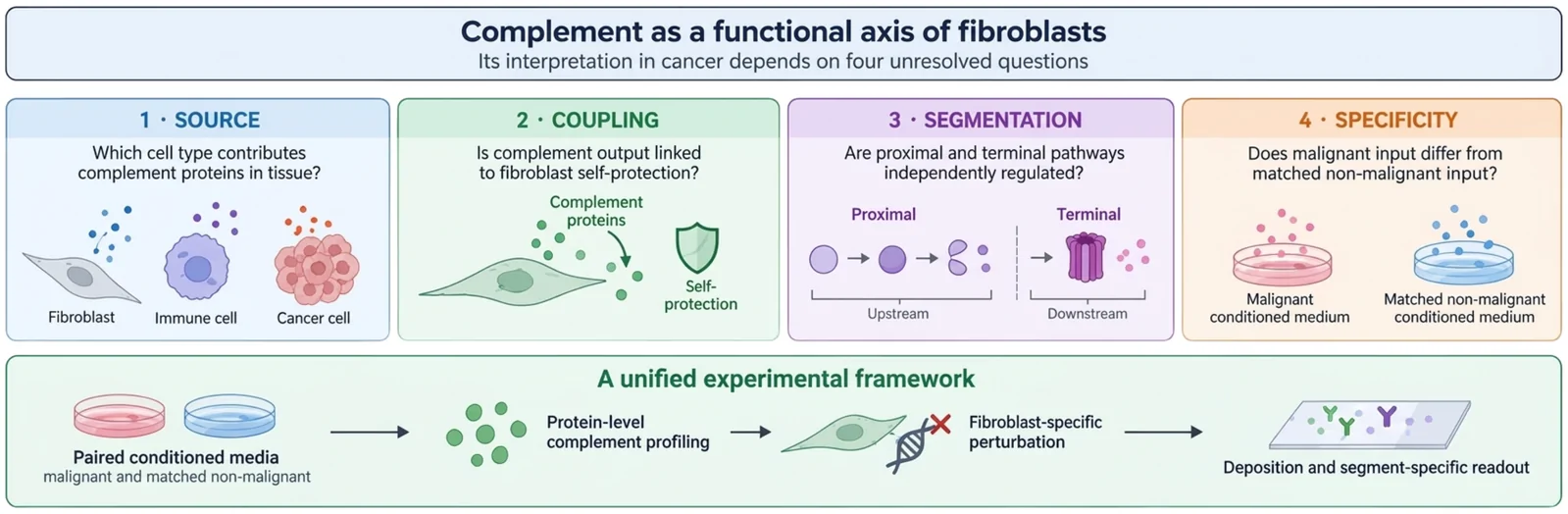
Complement is a real, perturbable functional dimension of fibroblasts, and its interpretation in cancer rests on four unresolved questions, three of which a single experiment can directly address. (1) Source attribution: single-cell and spatial data localise complement transcripts to CAFs and macrophages, but the net fibroblast contribution to complement protein in intact tissue, relative to tumour, myeloid, endothelial and circulating sources, is unquantified. (2) Production–protection coupling: production components and soluble regulators are co-expressed, but this has not been linked to surface C3/C5b-9 deposition, complement-mediated lysis or the membrane regulators CD46/CD55/CD59. (3) Cascade segmentation: signatures are proximal-biased although fibroblasts can synthesise terminal components, and whether this reflects active regulation or intrinsic bias is unresolved. (4) Malignancy specificity: tumour secretomes and IL-1 activate fibroblasts, but no study pairs a malignant with a matched non-malignant secretome under a complement readout. One design addresses three of these directly (production–protection coupling, cascade segmentation and malignancy specificity): paired malignant and non-malignant conditioned media on the same fibroblasts, complement quantified at the protein and secretion level (cell-free background subtracted), fibroblast-specific perturbation, and readout of surface deposition and per-segment abundance. This design establishes the fibroblast’s intrinsic capacity but cannot by itself quantify the net fibroblast contribution to complement in intact tissue (source attribution), which requires spatial, *in situ* protein quantification. Created in BioRender. Lei, K. (2026) https://BioRender.com/vy141pw.

The four questions are distinct rather than nested. Source attribution asks how to separate, quantitatively, the net fibroblast contribution to tissue complement from that of co-resident tumour, immune, endothelial and circulating sources. Coupling asks whether the inputs that drive complement production also build a functional self-protection programme. Composition asks which segments of the cascade the induction covers, in particular whether the recurrent bias toward the early (proximal) components over the late (terminal) ones reflects active regulation or merely an intrinsic detection bias. Specificity asks whether a malignant-cell secretome, rather than generic conditioned medium or tissue injury, is what triggers the response. **Figure 1** sets these out and, as this review argues, shows that a single experimental design can directly address three of them (coupling, composition and specificity) while constraining, though not fully resolving, source attribution, which ultimately requires *in situ* quantification in intact tissue.

The literature has raised these questions but not resolved them; this review sets out, for each, what the current evidence supports and what it cannot.

Existing reviews approach this topic from two directions but neither occupies its position. Reviews of complement in cancer are organised mostly by whole tumour or by immune-cell type, treating local and cell-intrinsic complement as a phenomenon shared across cell types rather than treating fibroblasts as an independent complement source unit (4, 5); reviews of the intracellular complosome (the pool of complement proteins acting inside cells in non-canonical, cell-autonomous roles) do include fibroblast-related content, but their frame is the intracellular complosome across immune cells, with fibroblasts as one part (6). This review differs by taking the source attribution, regulation and context dependence of fibroblast complement as its sole organising axis, and on that basis converging the field onto the four distinct, experimentally testable questions above.

Scope and literature selection. This is a critical, narrative review rather than a systematic review. I searched PubMed and Google Scholar (to 2026) using combinations of “complement”, “cancer-associated fibroblast”, “fibroblast”, individual component names (C3, C1r, C1s, C7, factor D, factor H) and “tumour microenvironment”, together with “single-cell” and “spatial transcriptomics”, and followed citations from the retrieved articles and from recent complement-in-cancer reviews. I prioritised studies that attribute complement to fibroblasts at the transcriptional, protein, secretion or functional level, alongside foundational non-cancer work on fibroblast complement biosynthesis and regulation. The counts in **Tables 1** and **2** are limited to the studies discussed here and are not an exhaustive systematic search.

**Table 1:** Semi-quantitative detection distribution of complement members in fibroblasts/CAFs within the scope of this review.

Complement member	Cascade segment	Detected within this review (study, cancer)	Breadth/note
C3	Proximal convergence	1–3, 7–12 (PDAC/breast/ESCC/uveal melanoma/gastric)	Broadest (~9 studies, ≥5 cancers)
C7	Terminal (soluble)	1, 3, 9, 11, 13–16	Broad (~8 studies)
CFD	Alternative-pathway initiation	1, 9, 11, 17, 18	Moderate (~5 studies; often with APOD)
C1R/C1S	Classical proximal proteases	CAF clusters: 1, 3, 12; also tumour-cell source: 19, 20	Few (~3 in cluster markers; mostly complement-focused studies)
CFH/CFI	Regulators	CFH: 1, 21 (FH protein); CFI: 1	Sparse
C4b	Classical	8	Single point
Terminal C5–C9 (MAC)	Terminal	Nearly absent from CAF cluster markers; seen only in metabolic-labelling studies (22, 23) and chemotherapy injury (24)	Near-absent → supports proximal bias

Counts are limited to the studies reviewed here and are not an exhaustive search; “detected” means the gene was reported as a fibroblast/CAF-associated complement signal in that study.

**Table 2:** One recurrent complement signature is distributed across seven fibroblast-state labels.

Study (cancer)	Assigned state	C1R	C1S	C4B	C3	CFD	CFH	CFI	C7
2 (PDAC)	iCAF				•				
15 (PDAC)	iCAF (IL6−)								•
8 (breast)	iCAF		•	•	•				
9 (ESCC)	iCAF1/iCAF2				•	•			•
11 (gastric/GEJ)	iCAF				•	•			•
1 (PDAC)	csCAF	•	•		•	•	•	•	•
7 (PDAC)	csCAF → early iCAF				•				
17 (HNSCC)	adipoCAF					•			
13 (pan-cancer)	progenitor CAF								•
14 (HCC)	CAF-C7								•
18 (chordoma)	cell-junction CAF					•			
3 (uveal melanoma)	fibroblast + macrophage	•	•		•				

Each row is a study reporting a complement-enriched fibroblast/CAF cluster; the “Assigned state” column gives the cell-state label that study applied, and a filled circle (•) marks each complement gene reported as a marker or feature of that cluster (a blank does not imply absence of expression, only that the gene was not listed). The same core signature (C3, C7, CFD, C1R/C1S) recurs across at least seven different state names, indicating that “complement = iCAF” is a naming convention rather than a validated attribution. Genes are ordered proximal → regulators → terminal.

Terminology. Much of the confusion in this field comes from sliding between levels of evidence, so I use a graded vocabulary. Complement-related or complement-enriched describes gene co-expression detected by transcriptomics, with no claim about protein. Complement-producing or complement-secreting is reserved for cases with nascent-protein or secretion evidence. Intracellular complement refers to cell-autonomous, non-canonical roles of complement proteins. Complement phenotype is an umbrella term for the recurrent transcriptional-and-protein state, used only where the level of evidence is stated. These terms are not interchangeable.

## 2 The complement system in brief

Complement is a cascade of soluble and membrane proteins activated through three routes (the classical, lectin and alternative pathways) that converge on cleavage of C3; cleavage of C5 then nucleates assembly of C5 through C9 into the terminal membrane-attack complex (MAC). Throughout this review, proximal denotes the recognition and C3-convertase components up to and including C3 (for example C1q, C1r, C1s, C2, C4, factor B, factor D and C3), and terminal denotes the C5–C9 components that form the MAC. Activation is held in check by soluble regulators such as factor H and C1 inhibitor and by membrane regulators such as CD46, CD55 and CD59. Several of these proteins additionally act inside cells in non-canonical, cell-autonomous roles, referred to collectively as the intracellular complement system or complosome. These complement genes should not be confused with the C1q/TNF-related proteins (CTRPs, encoded by C1QTNF genes), which share the C1q structural domain but are adiponectin-related secreted proteins rather than complement activators (25).

## 3 CAF state heterogeneity and attribution of the complement phenotype

CAFs comprise several plastic cell states maintained by microenvironmental signals. In pancreatic ductal adenocarcinoma (PDAC), Öhlund et al. (26) defined two spatially and phenotypically distinct subsets: myofibroblast-like CAFs (myCAFs), adjacent to tumour epithelium, αSMA-high, IL6-low and enriched for TGFβ-response genes; and inflammatory CAFs (iCAFs), further from the epithelium, αSMA-low, secreting IL6, IL11 and LIF and acting on tumour cells through STAT3. Biffi et al. (27) showed that tumour-derived IL-1 drives the iCAF state through NF-κB-induced autocrine LIF and JAK/STAT, antagonised by TGFβ, so that IL-1–NF-κB–LIF–JAK/STAT is a canonical iCAF axis in PDAC; Elyada et al. (28) added an antigen-presenting CAF, extending heterogeneity beyond the myCAF/iCAF dichotomy. Fibroblast activation protein (FAP), a widely used CAF surface marker, itself delineates functionally divergent stromal subsets (29); the complement-related phenotype discussed here is generally assigned to the iCAF axis rather than to FAP-high myofibroblastic CAFs. Two implications matter here. Because these are signal-maintained states, the iCAF label records the inputs a fibroblast is currently receiving rather than a fixed identity; and because the framework was built in PDAC, its extension to other cancers and to non-pancreatic fibroblasts such as dermal fibroblasts is not established. Although the literature routinely files the complement-related phenotype under iCAF, that correspondence is inconsistent across studies (26–28).

## 4 Cross-cancer distribution and state associations of the CAF complement signature

Complement-related genes are recurrently enriched in CAFs, yet the evidence that this represents a unified programme induced by the malignant secretome remains inadequate. Three lines of evidence point to different interpretations: first, studies disagree on which CAF subset the complement phenotype belongs to; second, trajectory inference and cell-atlas analyses suggest that complement expression may be a feature originally present in normal or early fibroblasts that is gradually attenuated or lost during tumour progression; third, tissue injury, anti-tumour therapy and chronic inflammation can also induce or maintain a functionally active complement system in fibroblasts. A pre-existing cell state, malignant-secretome induction, and injury or inflammatory responses can all generate this co-expression, and distinguishing the three mechanisms requires specific perturbation experiments (7, 24, 30).

### 4.1 A complement transcriptional signature reproduced across cancers

PDAC is currently the tumour type in which the complement-related CAF signature is most finely characterised. Chen et al. (1), from a single-cell atlas of 11 PDAC cases at different stages, defined a complement-secreting CAF (csCAF). This subset is characterised by expression of C3, C7, C1R, C1S, CFD, CFH and CFI, is distributed in the stroma immediately adjacent to malignant ducts, and is seen only in early lesions. csCAF is a subset name defined by transcriptional signature, and its secretion-level validation covers only some complement proteins. The histological evidence in that study mainly supports fibroblast localisation of C3 and the stage-dependent changes of C3 and C7. Croft et al. (2) further showed by spatial transcriptomics that in tumour-distal stromal regions C3 rises together with SFRP2, STAT3, IL6 and THY1, and validated the spatial distribution of C3 by immunohistochemistry; that study also performed pathway enrichment using a complement gene set but did not validate the other members individually. Thorlacius-Ussing et al. (15) assigned C7 to an iCAF transcriptional signature together with APOD, PTGDS and EGR1.

Similar complement expression patterns are also seen in breast and gastrointestinal tumours. Sebastian et al. (8) reported that the iCAF subset in the mouse 4T1 breast cancer model is enriched for C3, C4b, C1s1 and C1s2. Liu et al. (9) identified C3, C7 and CFD as marker genes of iCAF1 and iCAF2 in oesophageal squamous cell carcinoma (ESCC). In uveal melanoma, Zherka et al. (3) attributed the main transcriptional source of C1S, C1R and C3 to fibroblasts and macrophages. Here “source” still refers to expression attribution in single-cell data and cannot be equated with a direct measurement of protein production or secretory flux.

The complement genes repeatedly detected across cancers concentrate in a few members. CFD has been seen in head-and-neck squamous cell carcinoma, chordoma, ESCC and PDAC; C7 in ESCC, PDAC and pan-cancer atlases; and C3 has the broadest distribution. The classical-pathway proximal proteases C1R and C1S appear less often in studies whose main output is cluster marker genes; the csCAF gene list of Chen et al. (1) is one of the few to include them. C1R and C1S appear more often in studies dedicated to the complement system (3, 12). Whether a gene enters a study’s marker list therefore depends not only on its expression level but also on the research question, filtering thresholds and analysis strategy. Complement-high fibroblast clusters are also repeatedly accompanied by APOD, PTGDS, IGFBP6, FBLN1, SFRP2 and SFRP4. These markers overlap to some extent with cross-tissue steady-state or universal fibroblast states, but marker-level similarity alone is insufficient to define the corresponding CAFs as “resting” (31, 32).

The detection distribution of each complement member in fibroblasts/CAFs can be summarised semi-quantitatively as follows (**Table 1**).

### 4.2 Complement-high clusters span multiple CAF states

Complement is widely used as a categorisation marker for iCAFs, yet a unified marker panel and cross-dataset validation are still lacking. In existing studies the complement-high CAF cluster has been assigned to at least four different cell states (**Table 2**) (1, 8, 13, 17, 18).

Croft et al. (2), Sebastian et al. (8), Liu et al. (9) and Thorlacius-Ussing et al. (15) all assign the complement-high cluster to iCAFs. Other studies place it in different CAF subsets. Ho et al. (17) extracted 6,044 CAFs from single-cell data of 10 head-and-neck squamous cell carcinoma patients and divided them into four subsets; the smallest co-expressed CFD and APOD and was enriched for complement and lipid-metabolism pathways, and was therefore named adipoCAF, clearly separated in clustering from two inflammatory CAF subsets. Its subsequent hydrogel experiments aimed to recapitulate CAF heterogeneity *in vitro* and did not directly test the origin of the complement-high subset. Zheng and Guo (18) localised CFD in chordoma to a CAF enriched for cell-junction pathways, while defining another subset characterised by CCL4, HLA-DRA, IL1B and MMP9 as iCAF. The pan-cancer atlas of Chen et al. (13) listed C7, IGF1 and OGN as specific markers of three progenitor-CAF subtypes; its iCAF was defined by CCL2, CXCL12 and CXCL14, and its marker list did not include complement members. This indicates that complement genes were not incorporated into that study’s iCAF marker set, but does not license the inference that iCAFs express no complement at all.

Two non-mutually-exclusive explanations may account for this cross-study classification inconsistency. The first is methodological: studies use inconsistent clustering resolutions, marker-selection criteria and naming rules, so cell groups collectively called iCAF need not share the same biological composition. Thorlacius-Ussing et al. (15) reported that their C7-positive cluster does not express IL6, whereas another subset co-expresses IL6, LIF, HAS1 and CCL2, and on this basis proposed IL6-positive and IL6-negative iCAFs; however, that study is based on re-analysis of public PDAC single-cell data and marker-driven naming, without corresponding functional experiments. The second explanation has a biological basis: the complement programme and the inflammatory–chemokine programme may be two independently regulated transcriptional modules that co-occur in the same cell group only in some tissue contexts. Distinguishing their relative contributions will require targeted perturbation within the same experimental system; further clustering description provides limited discriminatory power. Any inference premised on “iCAFs express complement” should therefore state the clustering system and marker definition on which it relies.

This cross-study attribution is summarised in **Table 2**.

### 4.3 Complement features in normal and early fibroblasts

Several independent lines of evidence jointly point to a possibility: fibroblast complement expression is relatively high in normal tissue and early lesions and is gradually attenuated or lost during tumour progression. The most direct evidence comes from the stage description of csCAF. Chen et al. (1), when defining csCAF, already found that this subset appeared only in early PDAC. The team’s subsequent multi-omics work further showed that csCAF and iCAF overlap substantially in transcriptional signature and chromatin accessibility; the proportion of csCAF among all CAFs declines with pancreatic carcinogenesis; pseudotime analysis supports a potential csCAF→iCAF trajectory; and a higher csCAF proportion is associated with longer overall and recurrence-free survival. On this basis the authors interpreted csCAF as an iCAF subtype enriched in early lesions (7).

If this state trajectory is further validated, complement expression in CAFs can be understood as a fibroblast state not yet fully remodelled by the tumour microenvironment; a newly acquired tumour-induced programme would still require direct induction experiments. Three further observations are consistent with this interpretation. Sebastian et al. (8) also detected cells with iCAF and MHC-II CAF features in normal breast and pancreatic tissue, but that result concerns the whole subset phenotype and provides no complement-specific readout. Croft et al. (2) found that complement-expressing iCAFs are located mainly in tumour-distal stroma, and that expression of complement and inflammation-related genes is associated with longer survival; this survival association suggests that the function of complement-type CAFs may be context-dependent, with the causal direction still requiring experimental validation. Liu et al. (9) reported that complement-activation-characterised iCAF2 occupies a higher relative proportion in adjacent non-tumour tissue; this reflects a difference in subset composition and cannot be used to infer higher per-cell C3, C7 or CFD expression in adjacent tissue, nor to prove an anti-tumour function of the subset.

Placing these observations in the broader context of fibroblast biology reveals similar transcriptional associations. Buechler et al. (31) integrated ~230,000 fibroblasts from 17 tissues, identified two near-universal states, Pi16 and Col15a1, and proposed that these cells may constitute a reservoir for specialised and activated fibroblast states; when mapping human fibroblast states to mouse universal states, that study placed C3 and C7 among the correlated genes of the Dpt^+^Pi16^+^ and Dpt^+^Col15a1^+^ states, respectively. Gao et al. (32) further integrated 269,899 fibroblasts from 517 samples across 11 tissues and found that PI16-positive fibroblasts in adjacent non-tumour regions are associated with potential anti-tumour effects. Overall, a reproducible transcriptional association exists between complement expression and universal or steady-state fibroblast states; constitutive protein synthesis and anti-tumour causal function remain to be validated.

### 4.4 Injury, therapy and chronic inflammation induce and maintain complement responses

A separate line of evidence shows that under tissue injury, chemotherapy and chronic inflammation the fibroblast complement programme can be induced or maintained and produce experimentally perturbable functional effects. The key question is to define which exogenous inputs can sufficiently initiate this programme in which cellular contexts (24, 30, 33).

Monteran et al. (24), in a triple-negative breast cancer lung-metastasis model, found that doxorubicin chemotherapy drives lung fibroblasts to up-regulate a set of genes spanning proximal to terminal segments of the complement cascade, including C1ra, C1s, C2, C3, C4a, C5, C7, C8a and C8b, thereby promoting myeloid-derived suppressor cell recruitment and lung metastasis. The authors further compared three treatments in primary mouse lung fibroblasts: conditioned medium from untreated 4T1 cells, conditioned medium from doxorubicin-treated 4T1 cells, and direct doxorubicin exposure. Conditioned medium from drug-treated tumour cells induced C1ra, C2 and C3; direct drug action induced C5 and CFP; conditioned medium from untreated 4T1 cells failed to induce the complement factors tested. In this system, the untreated malignant secretome alone was insufficient to initiate the complement response measured, whereas the post-treatment tumour secretome and direct drug exposure each induced different complement components. This negative result applies only to the specific 4T1–mouse-lung-fibroblast system and its time and dose conditions, and cannot be extrapolated to the conclusion that all tumour secretomes are insufficient.

Human studies provide therapy-associated evidence but cannot yet directly establish therapy-induced causation. Zhang et al. (12) used spatial transcriptomics to compare 23 neoadjuvant-treated and 13 untreated PDAC cases and found higher expression of C1R, C1S, C3, C4B and C7 in the treated tumour microenvironment; single-nucleus RNA-seq further localised the C3 increase mainly to CAFs. Higher complement expression was also associated with more CD4-positive T cells, lower expression of immune-exhaustion genes, and longer overall survival. Because that study uses a cross-sectional comparison between different patient groups and lacks paired longitudinal sampling and experimental perturbation, its results support a “neoadjuvant-therapy-associated CAF complement state” but are insufficient on their own to prove that therapeutic injury is the direct upstream cause. At present this causal relationship is supported mainly by the experimental system of Monteran et al. (24).

Lin et al. (11) likewise studied the CAF complement phenotype in a treatment context, but the nature of its evidence needs to be delimited separately. That study reported the three-year follow-up of a single-arm phase II trial of neoadjuvant tislelizumab plus chemotherapy in locally advanced gastric and gastro-oesophageal junction adenocarcinoma. Single-cell sequencing was based on post-treatment surgical specimens from 7 patients, analysing 22,248 cells. iCAF was the largest CAF subset, characterised by CFD and expressing high C3 and C7; patients with higher iCAF scores showed stronger resistance to PD-1 blockade plus chemotherapy. The lack of paired pre-treatment single-cell data restricts the evidence to the existence of a CFD/C3/C7-high iCAF in post-treatment residual tumour; the therapy-induced origin of this state remains to be validated.

Studies outside cancer further show that fibroblast complement is a real, perturbable and functionally consequential dimension of biology. Friščić et al. (30), through genetic and pharmacological intervention, established a C3–C3aR–mTOR/HIF1α–NLRP3 axis for inflammatory tissue priming in synovial fibroblasts. Prasad et al. (33), in pulmonary hypertension, found that pulmonary-artery adventitial fibroblasts from patients and animal models up-regulate CFD, CFB and C3 and produce detectable C3a, C3b and C3d; shRNA knockdown of CFD reduced CFB activation and C3a generation and corrected the metabolic abnormality and pro-inflammatory phenotype. Both Friščić et al. (30) and Prasad et al. (33) provide perturbation evidence for fibroblast complement; the unique contribution of the latter is the pulmonary-hypertension context and the direct targeting of CFD.

### 4.5 Current evidentiary boundary of the CAF complement phenotype

Taken together, the CAF complement phenotype can be summarised as follows: complement expression is reproducibly observed across fibroblast datasets, but its CAF-subset attribution is not unified; a complement-related state exists in normal tissue and early lesions, and in PDAC the proportion of complement-high/csCAF-like cells appears to decline with progression, although the generality of this pattern across cancers remains uncertain; and tissue injury, therapy-associated contexts and chronic inflammation can also induce or maintain this state, with functional effects validated in some experimental systems.

Within the literature reviewed here, I identified no study that combined paired malignant and non-malignant conditioned media, normal fibroblasts, and a multi-component complement readout to directly demonstrate that the malignant secretome itself, relative to a matched non-malignant secretome, is sufficient to initiate the fibroblast complement programme. The negative result of Monteran et al. (24) in the 4T1–lung-fibroblast system provides an important boundary of applicability for this proposition, but it cannot be extrapolated into a cross-cancer conclusion.

## 5 Multi-source composition and quantitative attribution of the local complement milieu

Detecting complement transcripts in CAFs in single-cell data does not prove that fibroblasts are the main net source of complement protein in tumour tissue. The core of this section is a quantitative attribution question: of the complement protein and its activation products detected within tumour tissue, how much is locally synthesised by fibroblasts, and how much comes from malignant cells, myeloid and other immune cells, endothelial cells, or hepatic complement imported through the circulation. Current tumour research still relies mainly on single-cell and spatial transcriptomic localisation for cellular attribution of complement source. Although isolated CAFs and other fibroblast models have provided protein-expression, secretion and functional evidence, these results are still insufficient to quantify the relative contribution of each source in intact tissue (**Figure 2**) (10, 21).

**Figure 2:**
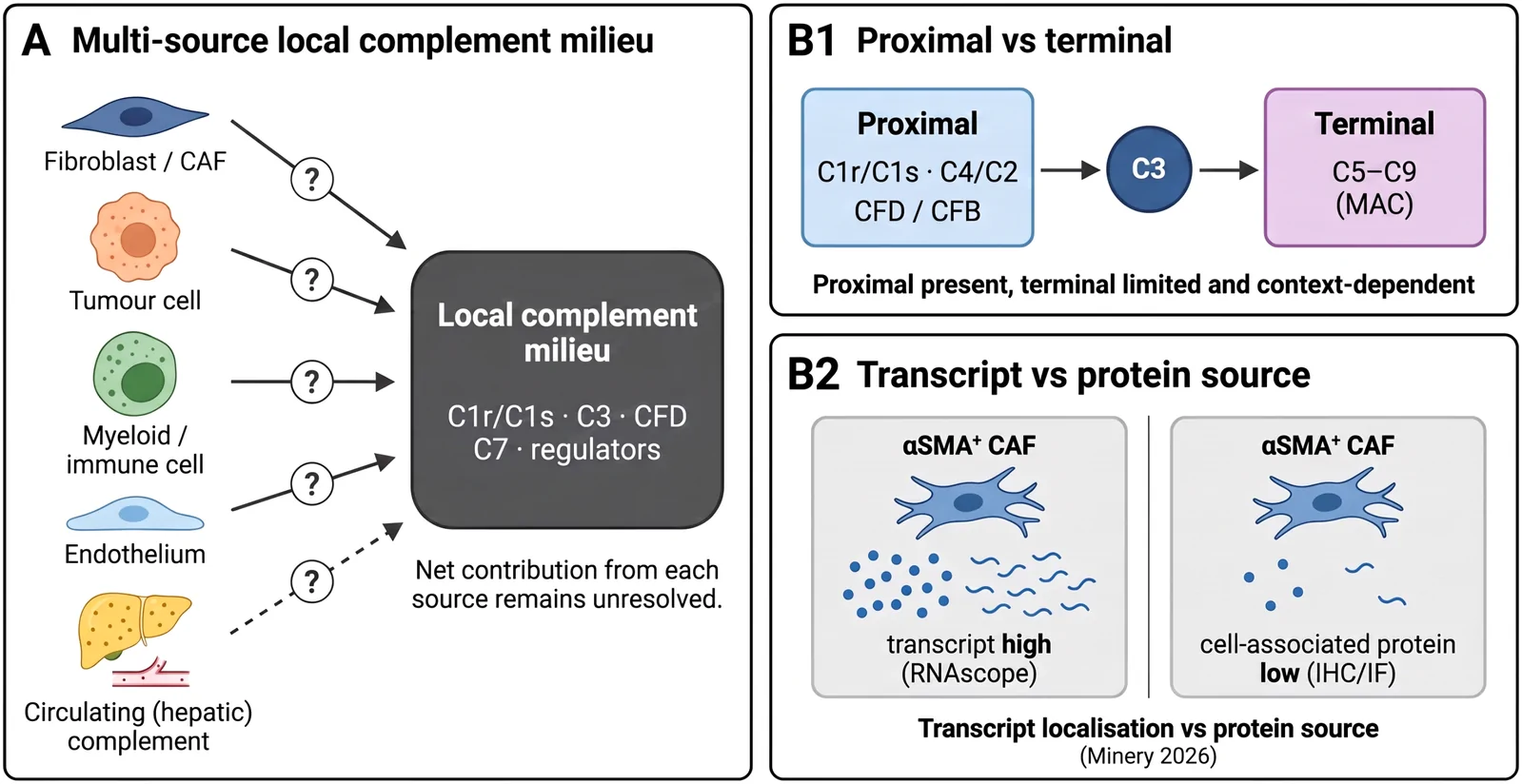
The local complement milieu is multi-source and the fibroblast contribution is not quantitatively attributed. **(A)** In intact tumour tissue, complement protein and its activation products may derive from fibroblasts/CAFs, malignant cells, myeloid and other immune cells, endothelium, and hepatic complement imported through the circulation (dashed arrow; not captured by single-cell RNA-sequencing). Question marks denote that the net contribution of each source to the local milieu is unresolved. **(B1)** Fibroblast complement output is biased towards proximal components, with limited and context-dependent terminal representation. **(B2)** High CAF transcript levels can coexist with low cell-associated protein signal, so transcript localisation does not establish protein source (19). The schematic frames open questions and does not assert quantitative contributions. Created in BioRender. Lei, K. (2026) https://BioRender.com/vy141pw.

Below, I evaluate in turn the evidence for each candidate source, analyse why current attribution methods cannot support a within-tissue proportional judgement, and further define the experimental design and quantitative evidence needed to resolve this problem.

### 5.1 Proximal complement contribution of malignant cells

The difficulty of complement source attribution begins with the fact that malignant cells themselves can express complement components overlapping with fibroblasts. Minery et al. (19) confirmed in triple-negative breast cancer that tumour cells intrinsically express C1R, C1S and C3, with evidence from both patient samples and *in vitro* experiments; these components can be further up-regulated by pro-inflammatory cytokines. The cell phenotype produced by knockdown of C1r or C1s could not be rescued by adding exogenous protein, supporting an intracellular mechanism of action. Therefore, in tissue or co-culture systems where tumour cells and fibroblasts coexist, detected proximal complement cannot be attributed to fibroblasts *a priori*.

Cutaneous squamous cell carcinoma likewise has tumour-epithelial-derived proximal complement. Riihilä et al. (20) found that cutaneous SCC tumour cells up-regulate C1r and C1s; knockdown of either reduced tumour-cell migration and suggested an action possibly independent of the complete classical cascade. The forward mechanistic chain C1s→MMP-9→invasion still lacks experimental support: after knockdown of C1r or C1s, MMP-9 expression rose rather than fell, and the study did not test its mediating role by MMP9 blockade or rescue. The study therefore supports high tumour-cell expression of C1r and C1s and their participation in migration; the current results are insufficient to prove invasion driven through MMP-9.

### 5.2 Immune, endothelial and circulating complement jointly compose the local pool

Besides tumour cells, fibroblasts face at least three further potential sources in the local complement milieu: immune cells, endothelial cells, and hepatic complement imported through the circulation.

Immune cells have the most complete evidence for complement synthesis. Lubbers et al. (34) systematically reviewed studies of complement production by immune cells, showing that monocytes, macrophages and many other immune cells can express and produce complement components. In tumour tissue this multi-source nature directly affects cellular attribution. Zherka et al. (3) attributed the main expression source of complement genes jointly to fibroblasts and macrophages. Measuring complement at the level of whole tissue or an entire stromal compartment therefore cannot automatically separate the fibroblast contribution from that of co-resident myeloid cells.

Endothelial cells constitute another local source. The four cell types compared by Lappin et al. (35) included umbilical-vein endothelial cells, and IFN-γ was found to induce overlapping but not identical patterns of complement-gene regulation in endothelial cells and skin fibroblasts. This means that an increase in stromal-compartment complement observed under inflammation cannot be assigned to a specific stromal cell source on spatial localisation alone.

Hepatic complement entering tissue through the circulation constitutes a third source, and is not captured by single-cell transcriptomics. Würzner (36) on the one hand listed fibroblasts as one extrahepatic source of C7 and on the other, based on C7 allotype analysis, summarised the major hepatic contribution to circulating C7. These two observations jointly show that extrahepatic synthetic capacity and the quantitative contribution of an extrahepatic source are two independent questions. Complement-protein measurements in tissue may also include components exuded from plasma, a source completely invisible to transcriptomic analysis.

Placing malignant cells, immune cells, endothelial cells and circulating input in one framework distinguishes four evidence levels: complement transcripts in CAFs; the secretory capacity of isolated CAFs or fibroblasts; the spatial localisation of complement protein in tissue; and the relative contribution of each source to the intact-tissue complement milieu. Modern tumour research has provided transcriptional, protein, secretory and functional evidence, but still lacks an experimental system that connects these levels and quantifies the contribution of different cellular sources (10, 19, 21).

### 5.3 The gap between CAF transcriptional enrichment and cell-associated protein signal

A further observation by Minery et al. (19) reveals the evidence gap this section must address. That study detected transcripts by RNAscope and protein by serial-section DAB immunohistochemistry and automated sequential multiplex immunofluorescence. The results showed that although C1R, C1S and C3 transcripts were relatively high in CAFs, the corresponding protein signal within or spatially associated with αSMA-positive CAFs was very low or undetectable.

This result must be interpreted cautiously in light of validation scope. RNAscope and immunohistochemistry showed one representative tumour and reported consistent results in two others; multiplex immunofluorescence showed one representative triple-negative breast cancer. The 22 breast cancers in the study belong to the single-cell sequencing cohort and cannot be treated as a protein-level validation sample size. The authors interpreted the low intra-CAF protein signal as due to secretion and retained the possibility that CAFs produce the corresponding protein. This observation establishes a spatial gap between transcript and cell-associated protein signal; transcript–protein uncoupling and re-distribution of protein source remain to be further validated (19).

Experiments on tumour cells in the same study addressed a different question: adding purified C1r, C1s or C3 protein, or a C1 inhibitor, failed to rescue the phenotypes of knockdown tumour cells, supporting an intracellular tumour-cell mechanism that cannot be extrapolated to conclude that fibroblasts do not produce or contribute these complement components (19).

Protein- and secretion-level evidence for CAF complement already exists in tumour research. Zhao et al. (10) detected C3 in patient-derived gastric-cancer CAFs and showed that p65 siRNA reduces C3 expression and secretion while weakening related downstream phenotypes. Rezola Artero et al. (21) detected FH protein in αSMA-positive CAFs of clear-cell renal cell carcinoma tissue and, in BJ cells and primary normal human dermal fibroblasts, assessed intracellular and secreted FH by immunoblot, ELISA and CFH knockdown. The core current gap concentrates on quantitative attribution in intact multi-source tumour tissue: within the studies reviewed here, the relative contribution of fibroblasts to the local complement milieu has not been resolved. Transcript and protein abundance are often concordant, and mRNA remains an efficient first-pass readout; the argument here is therefore narrower: protein-level confirmation matters specifically where transcript and protein diverge, as in the transcript-high, protein-low pattern reported by Minery et al. (19), and for the quantitative source question that transcripts alone do not address. Evidence from different complement components and different tumour contexts also does not substitute directly for one another.

### 5.4 Complementary evidence from nascent-protein measurement and transcriptional localisation

Whether fibroblasts can synthesise complement at the protein level was already answered clearly by early studies through direct nascent-protein measurement. Katz and Strunk (37), using ³^5^S-methionine metabolic labelling with immunoprecipitation, demonstrated that synovial fibroblast-like cells can synthesise C1r, C1s, C1 inhibitor, C2, C3, factor B and factor H. Katz et al. (38), using the same type of method, found that IL-6 increases factor B and C3 in human skin fibroblasts by roughly 40-fold and 15-fold respectively; Katz et al. (39) subsequently quantified the production of C3 and factor B under TNF-α alone. Rothman et al. (22) also detected nascent synthesis of C1s, C4, C3, C5, C6, C8 and C9 in WI-38 human lung fibroblasts.

The main difference between these early studies and contemporary single-cell work is the question answered. Metabolic labelling with immunoprecipitation directly measures nascent protein and thereby separates a cell’s own biosynthesis from serum input or cell-surface adsorption; single-cell transcriptomics has higher cell-type and spatial resolution but does not directly measure nascent protein synthesis. For the specific question of how much complement protein fibroblasts contribute in multi-source tumour tissue, transcriptional localisation improves cellular resolution and nascent-protein methods provide more direct synthesis evidence; the two method classes cover different dimensions, and evidence level must be judged separately in relation to the research question (19, 22, 37).

### 5.5 Experimental requirements for quantitative source attribution

Early metabolic-labelling experiments directly demonstrated that fibroblasts have the capacity to synthesise complement proteins, and Zhao et al. (10) and Rezola Artero et al. (21) added protein-expression, secretion and perturbation evidence for C3 or FH in tumour-associated systems. Current source attribution must solve a quantitative problem: in intact tissue where multiple sources coexist, how large is the relative contribution of fibroblasts to the local complement milieu. An experimental design to answer this should contain at least three elements: subtracting the complement background in cell-free medium or input conditioned medium; quantification at the protein and secretion level; and specific targeted perturbation of fibroblasts with measurement of its effect on the local complement abundance or flux in tissue.

## 6 Complement production, self-protection and segmental regulation of the cascade

This section examines in turn the co-regulation among complement genes, the coupling between complement production and cellular self-protection, and the differential regulation of proximal and terminal cascade components.

### 6.1 Stimulus- and context-dependent regulation of complement genes

Complement production shows component-specific regulation. In fibroblasts, different complement genes can respond differently, even oppositely, depending on upstream stimulus, dose, exposure time and cell source. This difference exists not only between proximal and terminal ends of the cascade but also within the proximal components (35, 37, 40).

The p65/RELA-dependent regulation of C3 reported by Zhao et al. (10) in established gastric-cancer CAFs provides relatively direct evidence for transcription-factor control of CAF complement output: p65 knockdown reduces C3 expression and secretion, and C3 knockdown weakens multiple CAF-promoted pro-tumour phenotypes. These results link CAF C3 output to an experimentally perturbable transcription factor, though that study addressed the maintenance of an already-established CAF state rather than its induction by the tumour-cell secretome, and did not test other components such as C1r and C1s.

Generalising these observations into a unified “NF-κB→complement” pathway would obscure substantial component- and context-specific regulatory heterogeneity. Katz and Strunk (37) found in the same synovial fibroblast-like cells that IFN-γ up-regulated all seven complement proteins tested, whereas lipopolysaccharide up-regulated only C3 and factor B. Katz and Strunk (41) then showed that IFN-γ, IFN-β1 and TNF exert distinct but partly overlapping induction of C1 inhibitor, C1r and C1s, indicating that the action of TNF involves both complement production and protective regulation. Katz et al. (39) further found that adding IL-13 on top of TNF further raised C3 yet lowered factor B. Lappin et al. (35) reported that IFN-γ up-regulated C2, factor B, C1 inhibitor, C4BP and factor H across four cell types while lowering C3 synthesis. Mitchell et al. (40) observed in primary fibroblasts and other systems that IFN-γ increased C3 and C4 mRNA and protein at 24 h, mainly through mRNA stabilisation. The two studies disagree on the direction of the C3 response, showing that cell source, stimulus conditions, exposure time and post-transcriptional regulation are all necessary experimental variables for interpreting complement expression changes.

Byun et al. (42) cloned ~2.15 kb of the 5′ flanking region of the mouse C1rA gene and, using reporter assays, serial deletion, site-directed mutagenesis and electrophoretic mobility-shift and supershift experiments, localised the region required for basal expression and IFN-γ response to −80 to −19, which contains an IRF-1 binding site. That study examined C1rA in mouse keratinocytes, did not perform chromatin-occupancy analysis, and did not test C1R, C1S or NF-κB subunits in human fibroblasts. The results support IRF-1 as a direct regulatory candidate for C1rA; the relationship of C1R, C1S and NF-κB in human fibroblasts remains to be tested.

Current evidence shows that a single signal can drive different complement components in opposite directions, and that the same complement gene can change its response direction with experimental context. C1rA already has IRF-1-site-level regulatory evidence, and the available evidence is consistent with multiple upstream regulatory inputs to the proximal complement proteases, although their relative contribution in human fibroblasts remains unresolved. A complete mechanistic statement must simultaneously specify the gene, cell context, stimulus dose and exposure time (35, 40–42).

### 6.2 Coupling of complement production with functional self-protection

Whether fibroblasts, while producing complement, simultaneously establish a self-defence programme with genuine protective effect is a stricter question than “whether production components and regulators are co-expressed.” Existing studies have simultaneously measured complement production components and soluble regulators in the same fibroblast system, but have not yet linked this co-regulation to cell-surface complement deposition, complement-mediated lysis, or changes in membrane-bound regulators (35, 37, 41).

That fibroblasts have complement self-protection capacity itself has fairly clear functional evidence. Friese et al. (43) showed that synovial fibroblasts can release FHL-1 and factor H, which bind to the cells’ own surface and reduce complement-mediated lysis; in that system IFN-γ and dexamethasone induced these two regulators, whereas TNF-α did not. Rezola Artero et al. (21) distinguished intracellular from secreted FH in BJ cells and primary dermal fibroblasts and found that intracellular FH supports proliferation and survival through a p53/Rb–E2F-related mechanism; this is an intracellular function of FH and does not directly concern defence against complement-mediated lysis, and therefore cannot be equated with the cell-surface protective mechanism described by Friese et al.

Katz and Strunk (37, 41) and Lappin et al. (35) have already, within one experimental framework, simultaneously measured production components such as C1r, C1s, C2, C3 and factor B and soluble regulators such as C1 inhibitor, factor H and C4BP. The functional questions still to be systematically tested include: whether this co-expression can functionally reduce surface C3 or C5b-9 deposition and complement-mediated lysis on fibroblasts; whether membrane-bound regulators CD46, CD55 and CD59 change in step with complement output; and whether perturbation of the same upstream pathway can causally alter complement production and protective capacity together. Friese et al. (43) demonstrated functional self-protection but did not systematically measure complement output in the same experiment. Current evidence already shows partial co-regulation of complement production and regulators; functional coupling to complement-protective capacity remains to be directly demonstrated.

### 6.3 Segmental expression and regulation of proximal and terminal complement

The literature shows that the complement cascade segment produced by fibroblasts varies with the experimental system. A “proximal present, terminal limited” pattern can arise jointly from intrinsic expression bias, active regulation or detection conditions, and current data do not distinguish these sources (22, 23, 37).

Several systems support a proximal-biased pattern. Katz and Strunk (37) showed that synovial fibroblast-like cells synthesise seven proximal or regulatory complement proteins, whereas C4 and C5 were undetectable. In adipose tissue and isolated adipocytes from BMI-discordant monozygotic twins, Kaye et al. (44) found up-regulation of early classical- and alternative-pathway genes but down-regulation of C5 and C6; because C8 and C9 were expressed at very low levels, these data do not show suppression of all terminal components. Zherka et al. (3) similarly reported preferential expression of early complement components in primary uveal melanoma.

Outside cancer, segmental patterns likewise depend on the comparison level: in an amyloid mouse model, Reichwald et al. (45) found that ageing and amyloid deposition raised proximal components (C1q, C3) more than terminal ones, with several terminal components below the quantification limit, a reminder that an apparent proximal bias can reflect detection sensitivity as much as active regulation, which bears directly on how the proximal-biased signatures reported in tumours should be read.

That fibroblasts can synthesise terminal components is likewise supported by direct experimental evidence. Rothman et al. (22), using ³^5^S-methionine metabolic incorporation with immunoprecipitation, showed that WI-38 human lung fibroblasts can nascently synthesise C1s, C4, C3, C5, C6, C8 and C9; of the C5–C9 components tested only C7 was not detected, though the WI-38 experiment did not cover all the regulators the study measured in A549 cells. Garred et al. (23) then reported that fibroblasts can synthesise C3 and C5–C9 and detected the activation-specific neoepitope of C9 on coated beads, providing functional evidence for terminal-complex assembly. Würzner (36) summarised multiple extrahepatic sources of C7 while noting, from transplantation and allotype studies, that circulating C7 still derives mainly from liver and bone marrow. These results provide direct evidence that extrahepatic C7 synthesis occurs but cannot be used to infer that the remaining circulating complement is mainly contributed by fibroblasts.

Recent tumour studies also use terminal components to define fibroblast subsets. Cheng et al. (14) named a CAF-C7 in hepatocellular carcinoma on the basis of high C7 and reported that this subset has a prognostic association opposite to CAF-FAP; however, its tissue cohort used FAP^+^VIM^+^ and PDGFRA^+^VIM^+^ as proxy markers and did not stratify survival groups directly on C7 protein. The study therefore established a subset–prognosis association; the causal function of C7 remains to be tested. Zhou et al. (16) reported that C7 is specifically expressed in prostate stromal cells, is directly transcriptionally regulated by the androgen receptor, and can inhibit tumour growth; but that study did not use lineage tracing to restrict the C7 source unambiguously to fibroblasts. Joseph et al. (46) distinguished, by *in situ* validation in normal and benign hyperplastic prostate, APOD-positive peri-epithelial fibroblasts from C7-positive interstitial fibroblasts. Monteran et al. (24) also found that chemotherapy injury can induce a cross-cascade expression profile including C5, C7, C8a and C8b, though that profile did not cover all terminal members.

That fibroblasts can nascently synthesise terminal components has been shown in several systems; the “proximal present, terminal absent” pattern seen in some studies may reflect specific regulation, detection sensitivity or system differences (22, 23).

There is already direct precedent for segment-selective regulation of the complement cascade. Pontoglio et al. (47) found in HNF1α-inactivated mice that C5 and C8A expression disappeared, C8B, C8G and C9 fell, while C3 and C7 were relatively preserved, proving that transcription-factor perturbation can produce segment-selective complement expression effects. Functionally, Jackson et al. (48), using a two-stage chemical carcinogenesis model, found that C3 deficiency reduced inflammation, epidermal hyperplasia and tumour burden, with an effect independent of the classical C5a or membrane-attack-complex pathways, showing that proximal and terminal complement functions can be decoupled.

Several more specific questions remain unsolved. First, which upstream signals produce which cascade-segment patterns in fibroblasts; the HNF1α studied by Pontoglio et al. (47) is a hepatocyte lineage-determining factor, so its results cannot be extrapolated directly to fibroblasts. Second, whether the tumour-cell secretome, chemotherapy injury and chronic inflammation induce reproducible and mutually distinct segmental patterns has not been compared in parallel on a unified experimental platform. Third, negative results require stricter reporting standards. Lubbers et al. (34) noted that “absence of production” is itself hard to prove experimentally; likewise, any non-detection of a terminal component should report both the detection limit and whether that component is reliably detectable at baseline.

## 7 Cellular and contextual dependence of malignant-secretome effects

Existing studies have shown that a tumour-cell secretome and IL-1 can push normal fibroblasts, including dermal fibroblasts, towards iCAF-like or other CAF-like states in pancreatic, colonic and skin models. What remains unresolved are three intersecting questions: whether malignant conditioned medium elicits a complement response distinct from that induced by matched non-malignant conditioned medium; whether a secretome derived from breast cancer or triple-negative breast cancer can produce a reproducible response in target-cell backgrounds such as the dermis; and whether complement is part of this malignant-secretome-specific induction (27, 49, 50).

Upstream induction of a CAF-like state itself has ample evidence. Díaz-Maroto et al. (49) found that tumour-cell-derived IL-1β can act on normal colonic fibroblasts and promote inflammatory CAF-like activation, and that blocking this signal prevents the transition. Biffi et al. (27) further established the IL1→NF-κB→JAK/STAT logic driving iCAF formation. Together, these studies provide experimental support for the feasibility of a tumour-derived inflammatory signal driving normal fibroblasts towards an iCAF-like state; complement has not yet been validated as a necessary component of that state.

These secretome studies bear on the specificity question only indirectly: they show that malignant secretomes can activate fibroblasts, not that they induce complement. Mazurkiewicz et al. (51) used conditioned media from four melanoma cell lines, Transwell co-culture and TGFβ treatment to push normal human dermal fibroblasts towards a CAF phenotype, with readouts including α-SMA, FAP-α, IL6, MMP2, MMP14, gelatin-degradation capacity and secretome changes, but the control was unconditioned plain medium. Forsthuber et al. (50) treated primary normal human dermal fibroblasts with conditioned media from melanoma and cutaneous SCC, using the fibroblasts’ own conditioned medium as control, and defined MMP1, MMP3, IL6, CXCL8, TGFB3 and IL1B as readouts of an iCAF-like phenotype. Both establish that a skin-derived malignant secretome can induce a CAF-like state; neither included complement in its readout, and neither included a matched non-malignant epithelial conditioned-medium source, so neither speaks to complement specificity.

Existing evidence constrains, but does not yet answer, the specificity of the malignant secretome. The relevant CAF state can be seen in normal tissue; the csCAF proportion declines with carcinogenesis; and high complement expression is associated with longer survival, so whether malignant and non-malignant conditioned media produce different effects must be determined by direct comparison and cannot be assumed as a premise (2, 7, 8). The same malignant secretome may also induce different CAF polarities depending on the target-cell background. Saxena et al. (52) found that MDA-MB-231 conditioned medium can drive mesenchymal stem cells towards myofibroblast-like CAFs, dependent on TGFβ signalling and matrix stiffness; this response direction differs from inflammatory iCAFs. Thus the malignant secretome can produce multiple CAF endpoints, with the specific direction depending on target-cell identity and microenvironmental conditions.

## 8 Synthesis: what each axis needs

### 8.1 Quantitative attribution in intact tissue

Multiple single-cell and spatial studies have computationally attributed complement transcripts to specific stromal cells. Zherka et al. (3) localised the main expression source of complement genes in uveal melanoma to fibroblasts and macrophages; Zhang et al. (12) localised the therapy-associated C3 increase mainly to CAFs; and the csCAF definition also rests on the transcriptional signature of fibroblasts (1, 7). However, these cellular attributions are still based mainly on transcriptional evidence. Zhao et al. (10) provided C3 protein-expression, secretion and perturbation evidence in isolated gastric-cancer CAFs; Rezola Artero et al. (21) localised CAF-associated FH protein in clear-cell renal cell carcinoma tissue and analysed FH secretion and intracellular mechanism in fibroblast models; and Minery et al. (19) observed high CAF complement transcripts but weak cell-associated protein signal in a limited number of tissue samples. The existing evidence is scattered across different tumour types, complement components and experimental levels and cannot yet quantify the net fibroblast contribution to the intact-tissue complement milieu. Resolving this requires, within one experimental system, background subtraction against cell-free medium and input conditioned medium, quantification of complement protein and secretion, and a tissue complement readout after fibroblast-specific perturbation.

### 8.2 Simultaneous testing of output and functional protection

Katz and Strunk (37, 41) and Lappin et al. (35) have already measured multiple complement production components together with soluble regulators such as C1 inhibitor, factor H and C4BP. What is missing is functional-coupling evidence: these studies have not linked co-expression to surface C3 or C5b-9 deposition on fibroblasts, complement-mediated lysis, or changes in the membrane-bound regulators CD46, CD55 and CD59. Friese et al. (43), although demonstrating that FHL-1 and factor H provide functional self-protection, did not systematically measure complement output in the same experiment; and the FH function validated by Rezola Artero et al. (21) mainly concerns intracellular cell-cycle regulation and cannot be equated with defence against complement-mediated lysis.

Resolving this requires, within the same fibroblast system, under identical stimulus conditions and on a continuous time axis, simultaneous measurement of multiple complement production components, soluble regulators and membrane-bound regulators, combined with perturbation of the same upstream pathway and a functional complement challenge, to test whether complement output and self-protection constitute a causally coupled programme.

### 8.3 Context dependence of the cascade-segment pattern

Existing studies provide two important precedents for segmental regulation of the complement cascade. Pontoglio et al. (47), in HNF1α-inactivated mice, observed selective decline of terminal complement genes, directly proving that transcription-factor perturbation can produce cascade-segment-specific expression effects. Jackson et al. (48), in a skin chemical-carcinogenesis model, found that the pro-tumour effect of C3 is independent of the C5a or membrane-attack-complex pathways, providing evidence for decoupling of proximal and terminal complement function. Together, these studies provide precedent for differential regulation and functional decoupling of proximal and terminal complement. The current unknown concentrates on the specific cascade-segment patterns fibroblasts form when facing the tumour-cell secretome, chemotherapy injury and chronic inflammation.

A unified experimental platform for comparing these conditions should also report the absolute abundance and detection limit of each complement component and analyse the different proximal members as independent regulatory units (34).

### 8.4 Direct comparison of malignant and non-malignant inputs

Among the four axes, the malignancy specificity of the complement phenotype carries the most evident evidentiary tension. Observations that constrain a tumour-specific interpretation include: the relevant fibroblast state is seen in normal tissue; csCAF declines with carcinogenesis and a higher proportion is associated with longer survival; complement-high cells are located in tumour-distal regions and associated with longer survival; iCAF2 has a higher proportion in adjacent tissue; and complement-related genes overlap with cross-tissue universal fibroblast states (2, 7–9, 31). On the other hand, chemotherapy injury, therapy-associated contexts, synovial inflammatory priming and pulmonary vascular inflammation can all induce or maintain a fibroblast complement response, proving that this programme is stress-responsive (12, 24, 30, 33).

These studies establish the tissue existence and stress-responsiveness of a complement-related state; the induction specificity of the malignant secretome relative to a matched non-malignant secretome still awaits direct comparison. Monteran et al. (24) observed that untreated 4T1 conditioned medium failed to induce the complement factors tested, but that negative result applies only to their specific system. Resolving this requires, in the same batch of target cells, a parallel comparison of matched malignant and non-malignant conditioned media and a non-tumour injury stimulus, reporting complement components and iCAF markers as distinct readouts.

## 9 Translational implications

The clinical significance of the complement-type fibroblast state is genuinely unresolved in direction. A higher complement/csCAF proportion is associated with longer overall and recurrence-free survival (2, 7, 9), suggesting fibroblast complement may be a favourable-prognosis stromal marker rather than a purely pro-tumour factor, contrasting with the commonly pro-tumour role of complement in cancer and underlining its context dependence. Two translational directions follow. As a biomarker, a complement/csCAF signature could help stratify early-stage disease if the favourable-prognosis association is confirmed at the protein and single-cell level, but only once its cellular source and directionality are established. As a target, systemic complement inhibitors (anti-C3 and anti-C5 agents already in clinical use for other indications) act on a pathway to which many cell types and the circulation contribute, so their net effect within a tumour is difficult to predict until the fibroblast contribution is quantified and its pro- versus anti-tumour valence resolved. Treatment response can be framed the same way: doxorubicin reshapes the lung-fibroblast complement programme (24) and neoadjuvant regimens associate with higher CAF-localised complement in pancreatic and gastric cancer (11, 12), raising the testable possibility that a complement/csCAF signature could act as a pharmacodynamic marker of treatment-induced stromal remodelling, though whether such changes drive or merely accompany response remains unresolved given current cross-sectional data. In both directions, quantitative source attribution is the gating experiment rather than an afterthought.

## 10 Conclusions: from cluster naming to a decisive experiment

Complement should be viewed not as a marker of a single CAF subtype, but as a real, reproducible and sometimes perdurable fibroblast functional dimension. What remains unresolved is the information needed to interpret or use it, the net fibroblast contribution to tissue complement, whether production is linked to self-protection, how cascade segments are regulated, and whether the malignant secretome is specifically responsible. The central lesson is that finer description alone, a new state label, atlas or marker list, cannot determine who produces the protein, whether it protects the cell, or whether malignancy is required.

The productive next step is a single, defined experiment (**Figure 1**). On one naïve fibroblast background, paired malignant and matched non-malignant conditioned media, together with a non-tumour injury control, are compared; complement is quantified at the protein and secretion level with cell-free background subtracted; fibroblast-specific perturbation tests causal contribution; and surface deposition, complement-mediated lysis and per-segment abundance are read out. Such a design speaks directly to coupling, segmentation and specificity at once and establishes whether fibroblasts have the intrinsic capacity in question; quantitative source attribution in intact tissue is the one axis this experiment does not settle on its own, requiring spatial, *in situ* protein measurement. Even so, it would move the field from cluster naming to mechanistic quantification (7, 10, 24, 31).

## Data Availability

No primary data were generated for this review. All data discussed are available in the cited, publicly available published studies; no new datasets or code were produced.
